# COVID-19 Misinformation and Infodemic in Rural Africa

**DOI:** 10.4269/ajtmh.20-1488

**Published:** 2020-12-30

**Authors:** Melody Okereke, Nelson Ashinedu Ukor, Lilian Muthoni Ngaruiya, Chikwe Mwansa, Samar Mohammed Alhaj, Isaac Olushola Ogunkola, Hadi Mohammed Jaber, Mashkur Abdulhamid Isa, Aniekan Ekpenyong, Don Eliseo Lucero-Prisno

**Affiliations:** 1 Faculty of Pharmaceutical Sciences, University of Ilorin, Ilorin, Nigeria;; 2 Faculty of Pharmaceutical Sciences, University of Port Harcourt, Port Harcourt, Nigeria;; 3 School of Pharmacy, Kenyatta University, Nairobi, Kenya;; 4 Michael Chilufya Sata School of Medicine, The Copperbelt University, Kitwe, Zambia;; 5 School of Medicine, Ahfad University for Women, Khartoum, Sudan;; 6 Department of Public Health, University of Calabar, Calabar, Nigeria;; 7 Faculty of Pharmaceutical Sciences, College of Medicine and Allied Health Sciences, University of Sierra Leone, Freetown, Sierra Leone;; 8 School of Health and Related Research, University of Sheffield, Sheffield, United Kingdom;; 9 Global Health Policy Unit, University of Edinburgh, Edinburg, United Kingdom;; 10 Department of Global Health and Development, London School of Hygiene and Tropical Medicine, London, United Kingdom

## Abstract

The world has witnessed rapid advancement and changes since the COVID-19 pandemic emerged in Wuhan, China. The significant changes experienced during these times remain unprecedented. The African continent has initiated significant responses to curb the spread of the pandemic. However, there is an increasing concern that rural Africa is facing serious challenges in their responses to the COVID-19 pandemic. This is due to the uncertainty if the populations are detached from or in synch with information on COVID-19. The findings reported here suggest that rural Africa is burdened with misinformation and infodemic regarding COVID-19 due to widespread misconceptions and anecdotal reports. It is, therefore, necessary to engage with community leaders to provide awareness campaigns in rural communities to ensure access to reliable information issued by local and international health authorities. It is pertinent to set up avenues that improve health literacy in communities in rural Africa as it is a major determinant of information assimilation.

## PERSPECTIVE

Since the COVID-19 pandemic emerged from Wuhan, China, the world has experienced rapid transformation and changes. The major changes that occurred during these times remain unprecedented. In light of these recent changes throughout the world, it is, however, becoming particularly difficult to ignore the existence of the COVID-19 pandemic in rural Africa alongside its resulting impact. There is an increasing concern that rural Africa is suffering several setbacks in their responses to the COVID-19 pandemic due to the uncertainty if the populations have access to accurate and evidence-based information on COVID-19. Although observations within the region have indicated a serious decline in access to information on COVID-19 in rural Africa, the mechanisms that underpin these observations are not fully understood. There is an urgent need to investigate the overall impact of the COVID-19 pandemic in Africa as well as provide novel insights on access to information on COVID-19 in rural Africa.

Although Africa was the last continent to be hit by the COVID-19 pandemic, the weak healthcare systems that are grossly underfunded in addition to the high burden of infectious and noncommunicable diseases (NCDs) make the continent extremely vulnerable,^1^ particularly the rural communities.^2^ From the first case of COVID-19 in Africa specifically in Egypt, on February 14, 2020, to date, there have been more than 1.5 million cumulative cases reported in all countries within the continent.^3^ Likewise, there have been more than one million cumulative recoveries, leaving the number of active cases amounting to more than 150,000, with about 35,000 deaths recorded.^3^ The gap between rural and urban Africa regarding the health workforce, infrastructure, and awareness is wide, making rural communities increasingly susceptible to the pandemic. A public health concern worth highlighting is the aged populations of rural communities who are often afflicted with comorbidities which make them more susceptible and prime candidates for COVID-19, particularly where precautionary measures are difficult to enforce. Such measures are often rejected because of strict cultural or religious beliefs followed by certain rural populations.

As a means of curtailing the community transmission of the COVID-19 pandemic, governments in various African countries adopted precautionary measures in accordance with the WHO guidelines including but not limited to lockdown, travel restrictions, and curfews. This has profoundly affected the rural communities, leading to a drop in agricultural output, incomes, and resultant food insecurity as well as poverty. In addition, regular access to health facilities is cut off, and in rural communities where a vast amount of the population suffers from NCDs such as hypertension and diabetes, the health of the inhabitants further deteriorates, leading to death from these comorbidities.^1^ In rural settings, access to social media and other information tools is limited to a selected few.^2^ On the other hand, many countries in the African region have poorly developed strategies and mechanisms for crisis and risk communication^4^ which presents several challenges to effective health communication in under-resourced settings in Africa. Noteworthily, these challenges translate to poor access to accurate and evidence-based information, and hence, misinformation and infodemic are widespread.^2^ Rural dwellers, unable to ascertain what is reliable and what is not, tend to believe whatever information is trending in their community. This creates an additional challenge of promoting precautionary measures and safety guidelines which will resultantly hamper the prevention efforts that have been made toward COVID-19 transmission in rural areas in Africa.

The first cases of COVID-19 in Africa were imported from other continents, and urban areas being the entry points, as international travelers were affected first. However, in a relatively short time span of the disease entry, the disease found its way to rural Africa because of delayed testing and poor contact tracing. These areas are the backbone of the continent’s agricultural outputs and where the larger percentage of the continents’ populations dwell. Key strategies were implemented to mitigate community spread in rural Africa. For instance, in Nigeria, physical distancing protocols characterized by restriction of movement were enacted. In Uganda, complete lockdowns and restriction of movement were enforced, and in Kenya, there was a complete ban on nonessential gatherings (not more than 15 people) for community events such as burials. Personal hygiene was also a key measure involving the use of hand sanitizers and handwashing areas. This approach witnessed the innovation and involvement of healthcare providers such as pharmacists in the production of sanitizers in Africa.^5^ Common areas of mass gatherings such as marketplaces and hospitals were required to be cleaned and fumigated at regular intervals to eliminate residual pathogens. Local leaders who are the moral compass and guides of the community were instrumental in capacity building and spreading awareness on the existence and disease control in rural settings. Although media outlets especially those broadcasting in local languages were used to spread information on the mode of transmission, prevention, and myths associated with the virus, these proactive efforts did not come without challenges.

Interventions and strategies to prevent widespread transmission of COVID-19 in African countries were based on evidence-based methods (handwashing, social distancing, testing, contact tracing, and lockdowns) adapted from areas first hit: Asian and European countries.^6^ Also used was the context from past pandemics and epidemics such as SARS and Ebola.^6^ In the early days of the pandemic, it was predicted that Africa will be severely hit. Although the number of infections in Africa remains comparatively lower than that in most other parts of the world, this assertion remains debatable. It is believed that low testing capacity and poor reporting foreshadow the reality of a wider spread.^6^ The proactive approach of government and health agencies in Africa to tackle COVID-19 in rural Africa has been met with a number of challenges that can be linked to misinformation or a lack of accurate health information. Factors such as poor living conditions, poor health literacy, ease of access to unambiguous information, influence of culture and religion, demography, and political instability greatly weigh in on the probability of prevention strategies to stay effective.^7^

## POOR LIVING CONDITIONS

People living in rural areas have little or no access to accurate health information and as such are likely unexposed to the reality of the risk of COVID-19.^7^ The paucity of accurate health information in rural areas is largely due to Internet unavailability and the inability to access television, social media—which serve as a medium for passing reliable health information by local and national health authorities. These act as a potential setback to governments’ effort to curb the spread of COVID-19.

### Low health literacy.

Globally, basic health literacy regarding COVID-19 is low because of limited access to health information. This is evident in low- and middle-income countries, especially in rural areas. As a result, individuals are unable to believe or reconcile the abstract notions of germ theory, invisible living organisms causing infections, infectivity despite being asymptomatic, and the concept of prophylaxis, vaccines, clinical trials, and placebo due to the lack of sufficient and reliable health information.^7^ These have posed as challenges hampering the improvement of health literacy regarding COVID-19 in rural Africa.

### Compromised access to accurate information.

A review of broadcast messages aimed at COVID-19 awareness in African countries revealed confounding in the messages, propagating doubt and hostility from the populace, as well as mistrust for health authorities.^8^ In an event of messages being unclear, people are often misinformed and also disregard other valuable information. The study^8^ identified that most awareness messages discouraged contact with animals. This, however, was not evidence-based and could affect how rural dwellers, particularly livestock farmers, perceived the information.

### Influence of culture and religion.

Globally, people attach a lot of sentiment and reverence for religious institutions and their leaders. If a religious leader misconstrues scientific facts, which is a common occurrence, there is a risk that the congregation would violate health-promoting behaviors. In Nigeria and Uganda, for instance, religious leaders have high credibility among their followers, and, as such, this serves as a means by which misinformation is propagated.^7^

### Political instability.

In areas plagued with unrest and political instability (war and civil strife), there is limited access to COVID-19 information.^9^ Also, the salience of COVID-19 risk is not as important as that of the instability already being experienced.^7^ There is as such no motivation to adhere to prevention strategies when information is available.

Some other factors that pose barriers to the implementation of prevention and control strategies in rural Africa include high population density,^7^ financial instability of the population, influence of culture, and inadequate research capacity, and cross-border dependence.^6^ Financial instability is often met by the need to defy lockdown to make money through the informal sector. Beliefs, culture, knowledge, and memory can also affect decision-making as regards obeying laid down prevention strategies. Furthermore, with a high population density, access to food, clean water, and sanitation is poor in rural Africa. Policies such as lockdown, therefore, meant that people with limited resources had to remain confined to small living spaces.

As already emphasized, there was a myriad of widespread misconceptions and misinformation about COVID-19 in several communities in rural Africa. For instance, in Sudan, people are convinced that the virus is yet to be a reality in the country and that it cannot spread in extreme temperatures^10^; thus, they do not adhere to any precautionary measures like social distancing or the use of nose masks. Others believe that the COVID-19 pandemic is a political conspiracy to receive aid and monetary support from health organizations.^10^ In Kenya, people in rural communities believe that the government uses the COVID-19 pandemic as an avenue to siphon donor funds,^11^ as well as a widespread belief that drinking alcoholic beverages can kill the virus.^11^ In Tanzania, it was reported that inhaling steam is highly effective against the coronavirus.^12^ In Nigeria, it is believed that the disease only affects people of higher socioeconomic status; thus, those residing in rural communities are immune to the disease. A distressing misconception in Africa of great public health concern is the belief that the genetic makeup of Africans provides strong immunity against the virus^13^; hence, there was no need to adhere to COVID-19 prevention protocols. These misconceptions, on the other hand, had a potential impact on COVID-19 management strategies in rural Africa, and this has led to setbacks in flattening the curve in most rural settings.

Africa’s healthcare system is known to be overstressed and underfunded,^14^ and this has made it easy for COVID-19 to wreak havoc on the system.^14^ It took 90 days for Africa to reach 100,000 coronavirus cases and about 19 days to exceed 200,000 cases in Africa.^3^ The disparity betwixt rural and urban Africa in terms of healthcare services has been described, and little or nothing has been done to bridge this gap. The misconceptions being spread has led many who need health services to stay back from health facilities with the perception of being immune to the disease.^13^ This in turn has made the proactive efforts being put by rural health workers futile and, as well, hampered disease management strategies amid the pandemic. A possible implication is that in the absence of evidence-based management strategies, this misinformation and widespread infodemic will result in a lot of deaths.

Following the speculations hovering in nearly every nation that the consumption of highly concentrated alcohol could disinfect and rid the body of the coronavirus, more than 2,000 cases of methanol poisoning and 264 deaths have been recorded in Iran.^15^ Even as the world is now in hope of potential vaccines for the coronavirus, a 6.4% drop in the United Kingdom and 2.4% drop in the United States regarding the willingness of citizens to take vaccines when there are available have been observed due to COVID-19 misinformation.^16^ Undoubtedly, these misinformation-related casualties have further intensified the COVID-19 crisis in rural Africa, and much is needed to be done to prevent a consequent second wave.

## CONCLUSION

Our article suggests that communities in rural Africa are detached from information on COVID-19 because of widespread misconceptions and anecdotal reports. The major challenges stem from the relatively poor access to information, culture and religion, political instability, living conditions, and poor health literacy. These findings suggest various realistic and novel courses of action. A reasonable intervention to tackle this issue will be to engage with community leaders to provide awareness campaigns in rural communities to ensure access to reliable information issued by local and international health authorities. It is necessary to set up avenues that improve health literacy as it is a major determinant of information assimilation. There is also a crucial need for an increased provision of resources (information and testing kits), training, and palliative to rural healthcare workers to aid their responses to COVID-19 and disease management skills. Furthermore, trained COVID-19 task force professionals should be deployed to rural settings to ease the pressure of the pandemic on rural healthcare workers and directly create an avenue for them to focus on disease management.
